# Emergency Stay Duration of Patients in Emergency Department of A Tertiary Care Hospital in Nepal: A Descriptive Cross-sectional Study

**DOI:** 10.31729/jnma.4806

**Published:** 2020-02-29

**Authors:** Prashant Simkhada, Shradha Acharya, Roshan Lama, Sujata Dahal, Nita Lohala, Ashish Thapa

**Affiliations:** 1 Kathmandu Medical College and Teaching Hospital, Sinamangal, Kathmandu, Nepal; 2 Tribhuban University Teaching Hospital, Maharajgunj, Kathmandu, Nepal; 3 Kathmandu University School of Medical Sciences, Dhulikhel, Nepal

**Keywords:** *emergency*, *length of stay*, *Nepal*

## Abstract

**Introduction:**

Emergency department of a hospital is responsible for providing medical and surgical care to patients arriving at the hospital in need of immediate care. Emergency department is not staffed or equipped to provide prolonged care. Duration of stay in the Emergency department directly affects the quality of patient care. Longer length of stay is associated with Emergency department overcrowding, decline in patient care, increased mortality and decreased patients satisfaction. The main aim of this study is to find the mean stay duration of patients in the emergency department of a tertiary care hospital in Nepal.

**Methods:**

This is a descriptive cross-sectional study which was conducted in a tertiary care teaching hospital from Jan 15, 2019 to Jan 30, 2019. Ethical clearance was obtained from Kathmandu Medical College- Institutional Review Committee. The calculated sample size was 587. Consecutive sampling technique was used. The data thus obtained was entered in SPSS version 20 and necessary calculations were done.

**Results:**

The mean emergency stay duration was obtained to be 3.18 hours at 95% confidence interval (2.98-3.38 hours) and standard deviation was 2.51 hours. Female had longer mean duration of stay (3.25 hours) compared to male (3.11 hours). The maximum length of stay was 15.3 hours. Three hundred ninety eight (67.8%) patients attending the emergency department were discharged right through the emergency department. Mean duration of stay was longest (5.06 hours) for the referral group.

**Conclusions:**

The mean stay duration in Emergency Department of tertiary care hospital in Nepal is getting shorter compared to similar study done previously.

## INTRODUCTION

Emergency stay is referred to the time elapsed from when a patient registers until the patient physically leaves the Emergency Department (ED). ED overcrowding has become a serious international health delivery problem.^1^ ED length of stay (LOS) is a widely accepted factor in ED crowding and is considered as a quantifiable surrogate marker for ED crowding and also a component of ED quality assurance monitoring.^2,3^

Prolonged ED stay is not good for patient care and it is shown to be associated with higher mortality rate and longer hospital length of stay among the boarded patients.^4,5^

Furthermore, ED overcrowding is associated with delay in diagnosis, treatment and hospital admissions, preventable medical errors, increased inpatient cases, reduced patient satisfaction regarding the care and decreased willingness to return to ED.^6,7,8^ A study done in ED of 3 tertiary care centers of Kathmandu in 1997 showed collective yearly volume of more than 100,000 visits.^9^ Thus, reducing the time a patient stays in ED may improve access of care to all the patients.

The aim of the study is to find out the average duration of stay of patient in ED and know the pattern of various consequence of the stay.

## METHODS

We conducted a descriptive cross-sectional study in Kathmandu Medical College and Teaching Hospital (KMTCH). The study was conducted for a duration of 15 days. Consecutive sampling technique was used. Study population included all the patients who presented to the Emergency department of KMTCH from 15th Jan 2019 to 30^th^ Jan 2019. Ethical clearance was obtained from Institutional review committee - KMCTH. The calculated sample size was 587.

Sample size was estimated as follows:

For continuous variable:

Sample size(n_1_)= Z^2^ (sd)^2^/e^2^

            = (1.96)^2^ × (2.5)^2^ / (0.3)^2^

            = 267

[Standard Deviation taken from Pilot study done in Emergency department of KMCTH]

Since consecutive sampling technique is used:

Actual sample size(n_2_)= 2 × n_1_

               =2 × 267

               =534

Considering 10% records with missing data:

Sample size(N) = n_2_ + 10% of n_2_

               = 534+53

            = 587

Relevant data was collected by retrospective chart review, required data thus obtained from ED records was entered in performa. Records with missing data were excluded. Emergency stay duration was calculated as the time in between the presentation of the patient till the patient leaves the Emergency department irrespective of the consequences of the stay.The consequences of the stay included in this study are- Treated cases, Discharged on request (DOR), Admission to ward, Admission to High Care Unit (HCU), Admission to Intensive Care Unit (ICU), Referral, Death or Obtunded.The data was entered and required descriptive statistics were obtained using SPSS version 20.

## RESULTS

The mean emergency stay duration was obtained to be 3.18 hours and standard deviation was 2.51 hours. The maximum length of stay was 15.3 hours (Table 1).

**Table 1:** Mean and Standard Deviation of length of stay.

	N	Maximum (hours)	Mean (hours)	Std. Deviation (hours)
Duration of stay	587	15.3	3.184	2.5144

The mean and standard deviation of stay for males was 3.11 hours and 2.60 hoursrespectively and mean and standard deviation of length of stay for female was 3.24 hours and 2.42 hours respectively ( Table 2).

**Table 2:** Duraton of stay for male and female.

Sex	Mean (hours)	N	Std. Deviation (hours)
Male	3.115	281	2.6064
Female	3.248	306	2.4295
Total	3.184	587	2.5144

**Figure 1:**
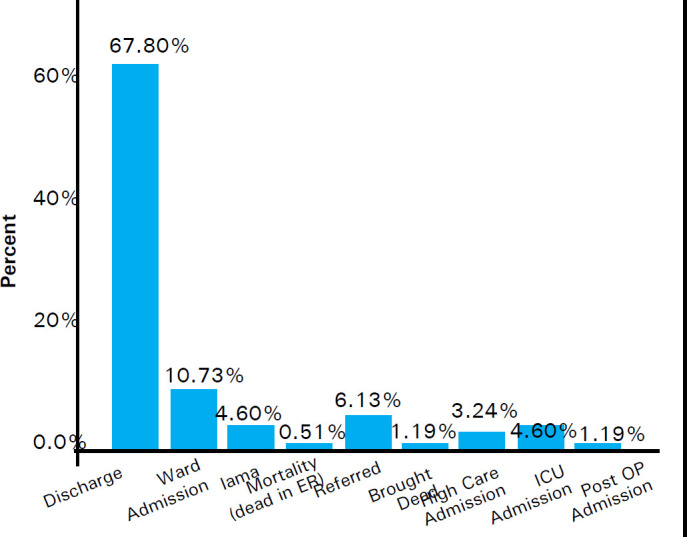
Various consequences of the Stay.

Out of 587 patients attending ED, 398 (67.8%) were discharged from the emergency department, 63(10.7%) were admitted to ward, 19 (3.2%) required high care admission, 27 (4.6%) required intensive care unit admission. Thirty six (6.1%) of patients were referred, 27 (4.6%) left against medical advise, 7 (1.2%) were admitted in the post- operative ward. Seven (1.2%) was brought dead in the emergency department and 3(0.5%) had mortality in the ED (Figure 1).

The mean stay and the standard deviation from mean length of stay of patients according to the outcomes are shown (Table 3).

**Table 3:** Mean stay and the standard deviation for different groups.

Consequence of Stay	Mean	Std. Deviaton
Discharge	2.855	2.4078
Ward Admission	3.935	2.1291
LAMA	3.744	2.1138
Mortality (dead in ER)	4.367	4.3386
Referred	5.061	3.2580
Brought Dead	2.129	2.0369
High Care Admission	3.742	2.9902
ICU Admission	2.872	2.0494
Post OP Admission	3.586	3.3229
Total	3.184	2.5144

## DISCUSSION

In our study, the mean duration of stay length was found to be 3.18 hours. This is less as compared to a study done by Acharya et al in which the mean duration of stay was found to be 18.1 hours.^10^ The low ER stay duration maybe because of the different emergency department set up and different working protocols. Patients needing ward and high care admission stayed longer than the patients who were discharged directly from ED which is similar in our study too. This result showed that there was a delay in admission of patient to ward and high care unit. As these patients didn’t receive needed inpatient evaluation and treatment while in ED so it may contribute to a long length of overall hospital stay.

A study done by Seyed et al showed 10.2% of patients visiting ED had prolonged LOS as compared to the maximum targeted length of stay of fewer than 6 hours introduced by the Iranian Ministry of Health and Medical Education (IMOH).^11^ Among these older age, lack of insurance support, higher number of ordered para-clinical tests were seen as important determinants of prolonged LOS. Factors inside ED such as delayed consult request, complicated cases, untimely admission, and crowding, were among the most frequent causes of prolonged LOS.

When ED are overwhelmed, their ability to respond to community emergencies and disasters may be compromised. Efforts aimed at reducing ED overcrowding and length of stay has been associated with increase in ED patient volume, decrease in number of patients who leave without being seen, reduction in costs and increased patients satisfaction.^12^ But various studies have shown that focusing only on a set of pre-specifed time rules may hamper the clinical priorities^13-15^.

In the study done by Meskin S et al, most of the patients seeking emergency care in their setting are young and present generally with acute trauma rather than chronic conditions.^9^ Interventions to decrease mortality and morbidity in emergency setting could increase life years saved. Thus, there is growing need for the establishment of well designed and responsive emergency care system in Nepal. However, the guidelines for management of emergency and disaster preparedness are yet to be streamlined by the concerned authorities. More research need to be done to find out the determinants influencing the ED LOS in our setting.

This is a single centered study and thus result lacks external validity. As the study is of short duration, it may not completely reflect the LOS and outcomes in other times of the year when the patients flow are very high or low.

## CONCLUSIONS

The ED LOS seems to be shortened at present. This might be a better sign that emergency department is getting robust at present time. But at the same time leaves us to think are the patients attending Emergency Departments getting adequate care during their stay in the ED.
